# KW2449 ameliorates collagen-induced arthritis by inhibiting RIPK1-dependent necroptosis

**DOI:** 10.3389/fimmu.2023.1135014

**Published:** 2023-03-13

**Authors:** Qiong Wang, Qinbin Ye, Xiaoyu Xi, Xiaoxue Cao, Xing Wang, Mengxiao Zhang, Yuan Xu, Tingting Deng, Xiaobing Deng, Guoqiang Zhang, Cheng Xiao

**Affiliations:** ^1^ Beijing University of Chinese Medicine, China-Japan Friendship Hospital Clinical Medicine, Beijing, China; ^2^ Institute of Clinical Medicine, China-Japan Friendship Hospital, Beijing, China; ^3^ Graduate School of Peking Union Medical College, Chinese Academy of Medical Sciences and Peking Union Medical College, Beijing, China; ^4^ Beijing Key Laboratory of Research of Chinese Medicine on Prevention and Treatment for Major Diseases, Experimental Research Center, China Academy of Chinese Medical Sciences, Beijing, China; ^5^ Department of Drug Discovery, Double-Crane Run Therapeutics, Beijing, China; ^6^ Department of Emergency, China-Japan Friendship Hospital, Beijing, China

**Keywords:** KW2449, rheumatoid arthritis, RIPK1, necroptosis, collagen-induced arthritis

## Abstract

**Objective:**

Necroptosis has recently been found to be associated with the pathogenesis of many autoimmune diseases, including rheumatoid arthritis (RA). This study was undertaken to explore the role of RIPK1-dependent necroptosis in the pathogenesis of RA and the potential new treatment options.

**Methods:**

The plasma levels of receptor-interacting protein kinase 1 (RIPK1) and mixed lineage kinase domain-like pseudokinase (MLKL) in 23 controls and 42 RA patients were detected by ELISA. Collagen-induced arthritis (CIA) rats were treated with KW2449 by gavage for 28 days. Arthritis index score, H&E staining, and Micro-CT analysis were used to evaluate joint inflammation. The levels of RIPK1-dependent necroptosis related proteins and inflammatory cytokines were detected by qRT-PCR, ELISA and Western blot, and the cell death morphology was detected by flow cytometry analysis and high-content imaging analysis.

**Results:**

The plasma levels of RIPK1 and MLKL in RA patients were higher than those in healthy people, and were positively correlated with the severity of RA. KW2449 could reduce joint swelling, joint bone destruction, tissue damage, and the plasma levels of inflammatory cytokines in CIA rats. Lipopolysaccharide combined with zVAD (LZ) could induce necroptosis in RAW 264.7 cells, which could be reduced by KW2449. RIPK1-dependent necroptosis related proteins and inflammatory factors increased after LZ induction and decreased after KW2449 treatment or knockdown of RIPK1.

**Conclusion:**

These findings suggest that the overexpression of RIPK1 is positively correlated with the severity of RA. KW2449, as a small molecule inhibitor targeting RIPK1, has the potential to be a therapeutic strategy for RA treatment by inhibiting RIPK1-dependent necroptosis.

## Introduction

Rheumatoid arthritis (RA) is a systemic autoimmune disease accompanied by chronic inflammation. It causes not only local joint inflammation but also cartilage destruction and bone erosion in severe cases (1). The incidence of RA has been reported to be as high as 0.4%, and it will continue to increase as the human lifespan grows (2, 3). However, even though many biomolecular mechanisms have been proposed, the current etiology of RA remains unclear, and targeted therapy is still difficult. It is still important to explore the pathogenesis of RA and new treatment methods. Recent studies have shown that RIPK1-dependent necroptosis plays an important role in autoimmune diseases, including RA (4, 5). Clinical studies have reported that mutations in the RIPK1 gene lead to its loss of function, which causes a variety of autoimmune inflammation conditions in patients, including arthritis. Moreover, studies have detected phosphorylated RIPK1 in the synovial tissue of RA patients (6, 7). These studies demonstrate the association of RIPK1 with RA. Therefore, we believe that RIPK1-dependent necroptosis may play a role in the pathogenesis of RA and is worth investigating.

Necroptosis is a type of regulated cell death triggered by perturbations of extracellular or intracellular homeostasis (8). It is characterized by the leakage of a large amount of intracellular contents and can elicit an immune response that triggers inflammation in other cells, leading to ongoing tissue damage (9) (10, 11). This is why necroptosis is considered to be an important mechanism in the pathogenesis of chronic autoimmune diseases such as RA (6, 12). In most cases, the triggering of necroptosis is heavily dependent on the signaling pathway formed by receptor-interacting protein kinase 1 (RIPK1), receptor-interacting protein kinase 3 (RIPK3) and mixed lineage kinase domain-like pseudokinase (MLKL) (8). As an upstream protein kinase, RIPK1 regulates not only necroptosis but also inflammatory and apoptotic signaling pathways (13, 14). Only when the action of caspase-8 is inhibited does phosphorylated RIPK1 combine with RIPK3 and form complex IIb, causing oligomerization and autophosphorylation of RIPK3 (15, 16). Phosphorylated RIPK3 recruits and phosphorylates MLKL, which causes cell membrane perforation and eventually leads to necroptosis (17). Overall, RIPK1 activation plays an important role in triggering necroptosis.

In this study, we identified an unclassified RIPK1 inhibitor (18), KW2449, and evaluated its therapeutic effects on RA using collagen-induced arthritis (CIA) rats, a classical RA animal model, and an *in vitro* necroptosis model induced by lipopolysaccharide (LPS) with the pancaspase inhibitor zVAD (19) in RAW 264.7 cells.

## Materials and methods

### Patients

Plasma samples were all obtained from the China-Japan Friendship Hospital. Twenty-three subjects without RA or other autoimmune diseases were enrolled as control samples (49.21 ± 16.12 years old on average). Forty-two patients were diagnosed with RA according to the 2010 American College of Rheumatology/European League Against Rheumatism (ACR/EULAR) criteria, with an average age of 53.52 ± 11.29 years (20). The RA patient group was divided into two groups based on their Disease Activity Score in 28 joints-Erythrocyte Sedimentation Rate (DAS28-ESR) (21, 22), the 5.1≥Activity≥2.6 group (n = 20) or the Activity >5.1 (n = 22) group. This study was reviewed and approved by the Ethics Committee of China-Japan Friendship Hospital with the ethical approval number of 2020-133-K86.

### Induction of CIA and treatments

Forty Sprague−Dawley (SD) rats (male, average body weight 200 ± 20 g, 6 weeks old) from the China Institute for Food and Drug Control, Beijing, China were used in this study. The rats were maintained under standard laboratory conditions at 23°C± 2°C with a 12-hour alternating light/dark cycle and were allowed ad libitum access to feed and water. After 3 days of adaptive feeding, arthritis was induced in rats by immunization with bovine type II collagen (Chondrex). Thirty rats were injected subcutaneously with a 50 μL emulsion of bovine collagen II and an equal volume of incomplete Freund’s adjuvant (Chondrex) at the tail root for primary immunization, and the remaining 10 rats were injected with an equal volume of normal saline. Seven days later, CIA rats were constructed by a secondary booster immunization using the same method. Four days after the secondary immunization, rats with different arthritis index (AI) scores (different severities) were equally assigned to each group, and the remaining rats without joint swelling were randomly assigned to each group. The drugs were administered by gavage. The blank control group and CIA group were given 10 mL purified water per kg of weight; the MTX group was given 1.5 mg MTX (Shanghai Sine Pharmaceutical Co) per kg of weight; and the KW2449 group was given 7 mg KW2449 (APExBIO) per kg of weight. All the above gavages were given once daily, and the rats were sacrificed after 28 days of continuous administration. The animal study was reviewed and approved by the Experimental Animal Ethics Committee of China-Japan Friendship Hospital with the ethical approval number of zryhyy21-21-11-02.

### Assessment of arthritis severity

#### Arthritis index score

After immunization, joint swelling was monitored every 3 days, and AI scores were recorded. Arthritis severity was expressed using an AI score ranging from 0 to 4 (23): 0=no swelling; 1=the little toe joint was mildly swollen; 2=the little toe joint and the heel were swollen; 3=the feet were all swollen below the ankle; 4=the whole joint, including the ankle, was swollen. The maximum AI score per rat was 16 (4 points×4 paws).

#### Hematoxylin and eosin staining

Joint tissue histopathology was evaluated by H&E staining. A histopathological score of 0 to 4 was scored according to the severity of cartilage and bone erosion, pannus and synovial hyperplasia. (0: no change; 1: mild; 2: moderate; 3: severe; 4: extremely severe) (23).

#### Micro-CT analysis

Bone erosion severity was assessed by micro-CT scanning of the ankle and paws using Skyscan1276 Micro-CT (Bruker, Belgium). 3D image reconstruction of the ankle and paw was performed using the matching software N-Recon. Then, bone volume (BV), bone surface area (BS), and BS/BV were analyzed in 3D by matching software CT-AN (24).

### Cell culture and treatments

The murine macrophage-like cell line RAW 264.7 was obtained from the ATCC. The cells were cultured in Dulbecco’s modified Eagle’s medium (Gibco) supplemented with 10% (v/v) fetal bovine plasma (Gibco) with 100 U/ml penicillin, 100 μg/ml streptomycin (Gibco) and 100 μg/ml Primocin (*In vivo*Gen) at 37°C in a humid atmosphere with 5% CO_2_. Then, RAW 264.7 cells were reseeded in 96-well or 6-well culture plates at densities of 1×10^5^ or 1×10^6^ cells/well and used for follow-up experiments. RAW 264.7 cells were stimulated with LPS (1 μg/ml) to establish a cell inflammation model and LPS (1 μg/ml) plus zVAD (20 μM; APExBIO) to establish a necroptosis model, with or without KW2449 (1 μM) or RIPA56 (1 μM; MedChemExpress LLC). The cell supernatant was collected 24 h after drug addition, and the cells were collected at the corresponding time points for detection.

### Cell viability assay

RAW 264.7 cells seeded in 96-well culture plates were incubated with different concentrations of KW2449 or DMSO for 24 h. Then, 10 µl Cell Counting Kit-8 (CCK-8; Dojindo) reagent was added to each well for an additional 1.5 h at 37° in the dark. The absorbance at 450 nm was measured with a microplate reader (Bio-Tek Instruments), and the cell viability was calculated using the formula in the manufacturer’s protocol.

### Quantitative real-time PCR

Total RNA was extracted using TRIzol (Invitrogen) and then reverse transcribed into cDNA. A Quant Studio 5 Real-Time PCR System (Thermo Fisher Scientific) was used for the qRT-PCR assay. The reaction conditions were set as 95 ° for 30 s followed by 40 cycles of 95 ° for 5 s and 60 ° for 34 s. The relative expression of genes was calculated using the 2^-△△Ct^ formula with β-actin as an internal control. The primers were synthesized by Sangon Biotech (Sangon Biotech).

### Nitric oxide assay

The NO content in the cell supernatant was detected by an NO assay kit (Beyotime). The reagent was added and incubated at room temperature for 10 to 20 min according to the instructions. The absorbance at 540 nm was immediately measured with a microplate reader (Bio-Tek Instruments).

### Enzyme-linked immunosorbent assay

The levels of TNF-α, IL-6 and IL-1β in the cell supernatant were detected by an ELISA kit (ABclonal) according to the manufacturer’s instructions. RIPK1 and MLKL levels in human plasma and cell supernatant were detected by an ELISA kit (Cloud-Clone Corp) according to the manufacturer’s instructions.

### Multiplex cytokine assays

IL-1α, IL-1β, IL-2, IL-4, IL-5, IL-6, IL-10, IL-12p70, IL-13, GM-CSF, G-CSF, IFN-γ, IL-17A/CTLA-8 and TNF-α levels in rat plasma were measured using a Th Complete 14-Plex Rat ProcartaPlex Panel (Thermo Fisher Scientific) according to the manufacturer’s instructions. Fluorescence was detected using a Luminex 200 system (Luminex Corporation) and analyzed using ProcartaPlex Analyst 1.0 software (Thermo Fisher Scientific). Only the cytokines that were above the detection limit were used in the analysis.

### Western blot

Cells were collected 6 h after drug intervention and lysed for 30 min on ice by RIPA lysis buffer with 1% protease and protein phosphatase inhibitor cocktail (Beyotime Biotechnology). Proteins were loaded onto 12% Super-PAGE Bis-Tris gels, separated by electrophoresis and then transferred onto PVDF membranes. Membranes were blocked with 5% skimmed milk for 1 h at room temperature, followed by incubation with antibodies against RIPK1, p-RIPK1, p-RIPK3, p-MLKL (Cell Signaling Technology, Cat# 3493, 31122, 91702, 37333), RIPK3, MLKL (Abcam, Cat# ab62344, ab243142) and β-actin (Santa Cruz Biotechnology Dallas, Cat# sc-47778) at 4 ° overnight. The proteins on the membranes were detected with a ChemiDoc XRS+ Gel Imaging System (Bio-Rad), after 2 hours of incubation with HRP-coupled goat anti-rabbit, goat anti-rat or goat anti-mouse secondary antibodies.

### Flow cytometry analysis of cell death

The cells in each group were incubated with fluorescein isothiocyanate-labelled annexin V (Annexin-V-FITC) and propidium iodide (PI) using an apoptosis kit according to the manufacturer’s instructions (BD Biosciences, Cat# 556547). After 15 min of incubation in the dark at room temperature, cell death was analyzed by flow cytometry (FACS; BD Biosciences).

### High-content imaging analysis of cell death

Cells in each group were double-stained with acridine orange/ethidium bromide (AO/EB) according to the instructions. The cell morphology was observed by an ImageXpress Micro Confocal High-Content Imaging System (Molecular Devices). A minimum of 200 cells per area were observed and counted separately by the following four morphologies: viable, early apoptotic, late-apoptotic and necroptotic cells (25).

### Small interfering RNA transfection

Ripk1 siRNA and negative control siRNA were obtained from Gene Pharma (Suzhou, China). The sequences of Ripk1-mus-1633 siRNA were 5′- GCAGAGAGCUCGUGAGAAUTT-3′ (forward) and 5′- AUUCUCACGAGCUCUCUGCTT-3′ (reverse). The cells were stimulated with LPS (1 μg/ml) plus zVAD (20 μM) (LZ) 24 hours after transfection by Lipofectamine 3000 (Invitrogen Life Technologies), and the expression of RIPK1 was measured by qRT−PCR after another 24 hours. Cells were stimulated with LZ 72 hours after transfection, and RIPK1 expression was measured by WB 6 hours later.

### Statistical analysis

GraphPad Prism software (version 9.0) was used for statistical analyses. All data are presented as the mean ± SEM. The two-tailed Student’s t test was used for comparisons between two groups, and Welch’s T test was used for nonnormally distributed data. One-way ANOVA was used to compare the data between three or more groups, and Brown-Forsythe and Welch ANOVA tests were used for nonnormal distributions. Statistical significance was considered when the P value < 0.05.

## Results

### KW2449 shows therapeutic effects on CIA rats

**Figure 1:**
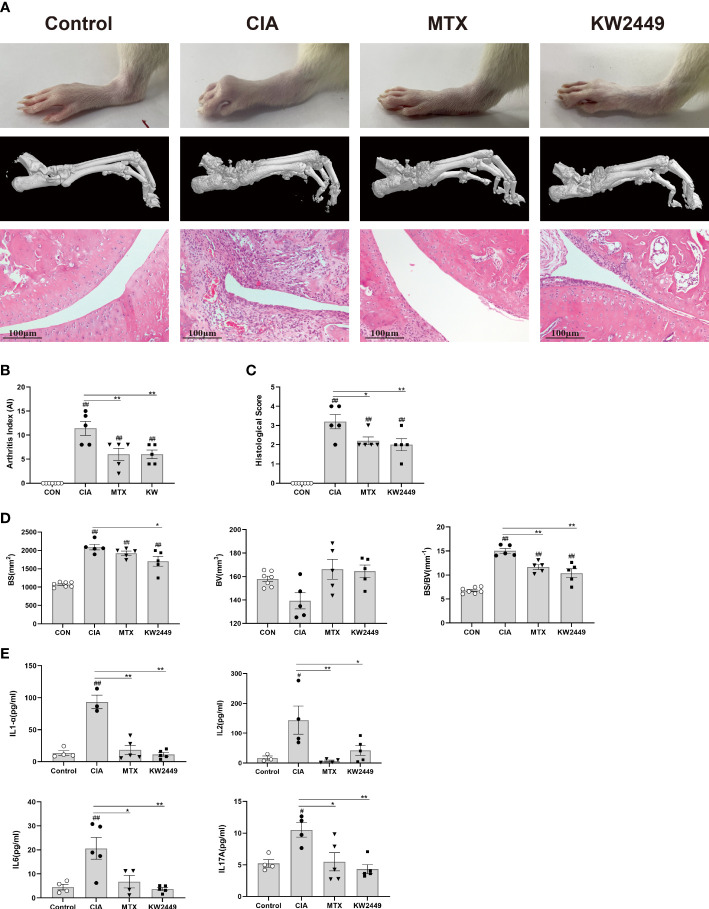
KW2449 reduced inflammation, joint tissue damage and joint bone destruction in CIA rats. **(A)** Representative ankle images, micro-CT images of ankle joints and HE-stained ankle tissue images of each group. Original magnification: 100×, scale bars = 100 μm. **(B)** Arthritis index (AI) scores of each group after treatment. **(C)** Histological score of each group. **(D)** The bone surface area (BS), bone volume (BV), and BS/BV values of ankle joints in each group. E Plasma levels of inflammatory cytokines IL-17A, IL-1α, IL-2 and IL-6 in each group. Means ± SEs, n=5, ^#^ *P <*0.05, ^##^ *P <*0.01, vs. control, *P <0.05, **P <0.01, vs. CIA.

To determine the efficacy of KW2449 on RA, methotrexate (MTX), which has been considered effective in the clinical treatment of RA for many years, was selected as a positive control drug (26, 27). After 28 days of intragastric administration, we found that both KW2449 and MTX showed obvious therapeutic effects on CIA rats, not only improving the severity of joint swelling and reducing the arthritis score (**Figures 1A, B**) but also reducing the release of inflammatory cytokines in the plasma of CIA rats, such as interleukin-17A (IL-17A), interleukin-1α (IL-1α), interleukin-2 (IL-2) and interleukin-6 (IL-6) (p<0.05; p<0.01) (**Figure 1E**). Histopathological results showed that compared with the blank control group, the ankle joint structure of rats in the CIA group was damaged, and the erosion of cartilage and bone was serious, accompanied by a large amount of pannus formation, synovial hyperplasia and inflammatory cell infiltration. After KW2449 or MTX treatment, these pathological characteristics improved, and the histopathological score decreased significantly (p<0.05) (**Figures 1A, C**). To further evaluate bone destruction, we performed three-dimensional reconstruction of the rat ankle joint by microcomputed tomography (micro-CT), which showed significant bone destruction and increased BS/BV values (p<0.01) in the CIA group, which were improved after treatment with KW2449 or MTX (p<0.01) (**Figures 1A, D**). This showed a protective effect in both treatment groups. These results indicate that KW2449 can inhibit the progression of CIA rats. It can alleviate joint bone destruction and joint tissue damage in CIA rats and reduce the release of plasma inflammatory cytokines. We next explored the mechanism by which KW2449 exerted its therapeutic effects on CIA rats.

### The levels of RIPK1 and MLKL increased in the plasma of RA patients

**Figure 2:**
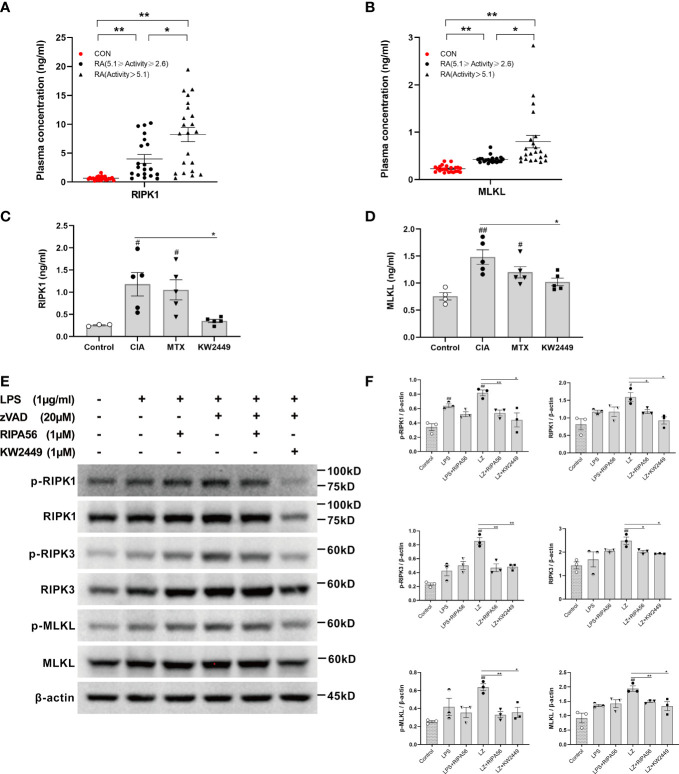
The level of RIPK1-mediated necroptosis-related proteins in RA and the effect of KW2449 on RIPK1. **(A, B)** ELISA analysis of RIPK1 and MLKL protein levels in the plasma of healthy people (n = 23) and RA patients. RA patients were grouped by the Disease Activity Score in 28 joints-Erythrocyte Sedimentation Rate (DAS28-ESR), 5.1≥Activity≥2.6 (n = 20), Activity >5.1 (n = 22). Means ± SEs, *P <0.05, **P <0.01.**(C, D*)*** ELISA analysis of RIPK1 and MLKL protein levels in the plasma of rats in each group. Means ± SEs, n=5, ^#^ *P <*0.05, ^##^ *P <*0.01, vs. Control, *P <0.05, vs. CIA. **(E, F)** p-RIPK1, RIPK1, p-RIPK3, RIPK3, p-MLKL and MLKL expression in RAW 264.7 cells was detected by Western blot. Means ± SEs, n=3, ^#^ *P <*0.05, ^##^ *P <*0.01, vs. control, *P <0.05, **P <0.01, vs. LZ.

It has been reported that RIPK1-mediated necroptosis is associated with autoimmune diseases (4–7). To verify whether RIPK1-mediated necroptosis also has the same effect in RA patients, we detected and compared the plasma levels of RIPK1 and MLKL in RA patients and healthy controls. We found that the plasma levels of RIPK1 and MLKL in RA patients were significantly higher than those in healthy people, and the plasma levels of RIPK1 and MLKL in the severe group were higher than those in the mild-moderate group (p<0.05; p<0.01) (**Figures 2A, B**). These results suggest that the RIPK1-mediated necroptosis pathway plays a role in the pathogenesis of RA and is positively correlated with disease severity.

### KW2449 exerts its therapeutic effect on CIA rats by inhibiting RIPK1

We detected the plasma levels of RIPK1 and MLKL in each group of rats and found that their levels in the CIA group were significantly higher than those in the control group (p<0.05; p<0.01) (**Figures 2C, D**). This suggests that RIPK1-mediated necroptosis plays an important role in the pathogenesis of CIA. The levels of RIPK1 and MLKL decreased to varying degrees after treatment with KW2449 (p<0.05), but there was no significant change in the MTX treatment group (**Figures 2C, D**), suggesting that KW2449, but not MTX, had an inhibitory effect on RIPK1.

The proteins involved in the RIPK1-mediated necroptosis pathway were examined in the LPS-induced inflammation model and LZ-induced necroptosis model *in vitro*. To clarify the inhibitory effect of KW2449 on RIPK1, the type III RIPK1 inhibitor RIPA56 was selected as the positive control in the cell experiments (18, 28). The results showed that the levels of proteins involved in the RIPK1-mediated necroptosis pathway were slightly increased after LPS induction, but the difference was not statistically significant, and there was no significant change after treatment with RIPA56 or KW2449 (**Figures 2E, F**). However, after LZ induction, the levels of RIPK1, RIPK3 and MLKL increased significantly (p<0.05; p<0.01), accompanied by increased levels of p-RIPK1, p-RIPK3 and p-MLKL (p<0.05; p<0.01), which were decreased to varying degrees after treatment with RIPA56 or KW2449 (p<0.05; p<0.01) (**Figures 2E, F**). This observation illustrates the inhibitory effect of KW2449 on the RIPK1-mediated necroptosis pathway. Moreover, these results suggest that necroptosis cannot be induced by LPS stimulation alone and that a caspase inhibitor, such as zVAD, must be added. Based on the *in vivo* and *in vitro* experiments, it is reasonable to believe that the mechanism by which KW2449 exerts its therapeutic effect on CIA rats is the inhibition of RIPK1-mediated necroptosis.

### KW2449 reduces the release of inflammatory cytokines by inhibiting RIPK1-mediated necroptosis

**Figure 3:**
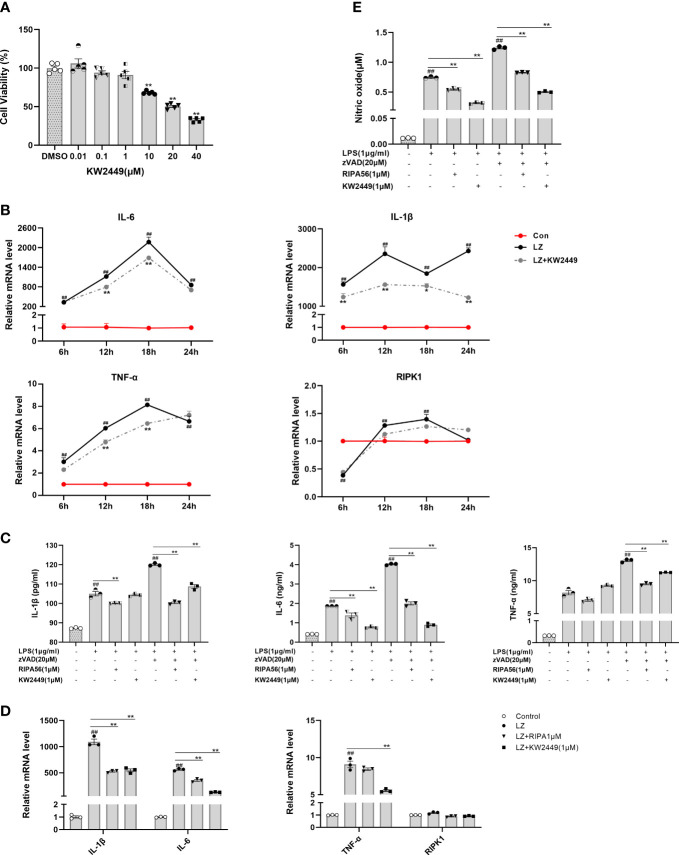
KW2449 reduced inflammation in LZ-induced necroptosis. **(A)** Cell viability in RAW 264.7 cells was determined at different concentrations of KW2449 by CCK-8 assay. Means ± SEs, n=5, *P <0.05, **P <0.01, vs. DMSO. **(B)** The effects of KW2449 on IL-6, IL-1β, TNF-α and RIPK1 at different time points in RAW 264.7 cells induced by LZ were measured by qRT−PCR. **(C)** The effect of KW2449 on IL-6, IL-1β and TNF-α for 24 hours in RAW 264.7 cells induced by LZ was detected by ELISA. **(D)** The effect of KW2449 on IL-6, IL-1β, TNF-α and RIPK1 for 12 hours in RAW 264.7 cells induced by LZ was measured by qRT−PCR. **(E)** The effect of KW2449 on NO for 24 hours in RAW 264.7 cells induced by LZ was detected by an NO assay kit. Means ± SEs, n=3, ^##^ *P <*0.01, vs. control, *P <0.05, **P <0.01.

RIPK1 is involved in the regulation of multiple signaling pathways, such as inflammation, apoptosis and necroptosis (13, 14). Therefore, for the previously detected inhibitory effect of KW2449 on inflammation, it is difficult to tell whether it acts directly on RIPK1-mediated inflammation or by acting on necroptosis to reduce the release of inflammatory cytokines. To this end, we examined the changes in inflammatory cytokines in the LPS-induced inflammation model and LZ-induced necroptosis model after KW2449 treatment. First, we found that KW2449 was non-toxic up to 1 µM, but began to show toxicity to cells at 10 µM by CCK-8 assay, so we selected 1 µM for subsequent cell experiments (**Figure 3A**). Second, the efficacy against LZ-induced necroptosis was detected at 6 h, 12 h, 18 h and 24 h (**Figure 3B**). The results showed that KW2449 had a better inhibitory effect on RIPK1 at 12 h and 18 h and reduced the expression of inflammatory cytokines, such as tumor necrosis factor-α (TNF-α), IL-6 and interleukin-1β (IL-1β), at the mRNA level (**Figure 3B**). In parallel, we also examined the effects of KW2449 or RIPA56 on LZ-induced necroptosis at 12 h and found that both had similar inhibitory effects on inflammatory cytokines at the mRNA level (**Figure 3D**). Although the changes in RIPK1 at the mRNA level after LZ stimulation were small, they had a clear effect on its protein level and the expression of inflammatory cytokines (**Figures 2E, F**, **3C, D**). Finally, by measuring the expression of inflammatory cytokines in the cell supernatant, we found that both RIPA56 and KW2449 showed weak inhibitory effects on inflammatory cytokines in the LPS-induced inflammation model, and only when zVAD was added to induce necroptosis did they show significant inhibitory effects (p<0.01) (**Figure 3C**). In addition, we found that KW2449 or RIPA56 reduced the release of nitric oxide (NO), another inflammation-related marker, in both LPS-induced and LZ-induced models, although the effect was more pronounced in the LZ-induced model (p<0.01) (**Figure 3E**), which may be due to its complex network relationship (29). Collectively, we found that KW2449 exerted a stronger anti-inflammatory effect on LZ-induced necroptosis than on LPS-induced inflammation in RAW 264.7 cells. This suggests that the mechanism by which KW2449 reduces inflammatory cytokines is mainly through the inhibition of RIPK1-mediated necroptosis rather than a direct effect.

### KW2449 protected RAW 264.7 cells from necroptosis but not apoptosis induced by LZ

**Figure 4:**
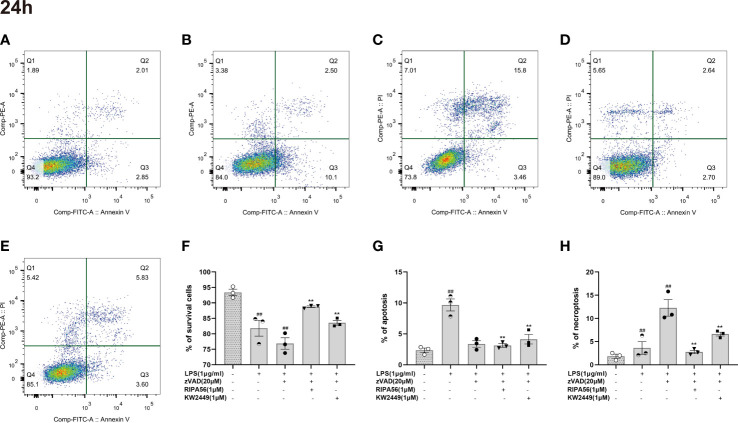
KW2449 reduced LZ-induced necroptosis in RAW 264.7 cells, as detected by flow cytometry using Annexin V-FITC/PI staining. **(A-E)** Representative graphs of cell death (24 h). **(A)** Control; B: LPS (1 μg/ml); C: LPS (1 μg/ml) + zVAD (20 μM); D: LPS (1 μg/ml) + zVAD (20 μM) + RIPA56 (1 μM); E: LPS (1 μg/ml) + zVAD (20 μM) + KW2449 (1 μM). **(F)** The population of A-P- for cell survival. **(G)** The population of A+P– for apoptosis. **(H)** The population of A+P+ for necroptosis. Means ± SEs, n=3, ^##^ *P <*0.01, vs. control, **P <0.01, vs. LZ.

At present, many types of regulated cell death have been found (8), and whether KW2449 acts on LZ-induced necroptosis or apoptosis was further verified in our experiments by cell staining and morphological observation. To avoid natural cell apoptosis in the experiment as much as possible, we chose to detect and analyze the cells 24 h rather than 48 h after the addition of the drug. Cells were stained with Annexin V-FITC/PI, and the proportion of different cell death types in each group was detected using flow cytometry (**Figures 4A–E**). We identified AV+PI- cells as apoptotic cells and AV+PI+ cells as necroptotic cells and counted them. We found that the main cause of cell death after LPS stimulation alone was apoptosis, while LZ stimulation produced more necroptosis. After treatment with KW2449 or RIPA56, the proportion of apoptotic cells did not change significantly, but the number of necroptotic cells was significantly reduced (p<0.01) (**Figures 4F–H**).

**Figure 5:**
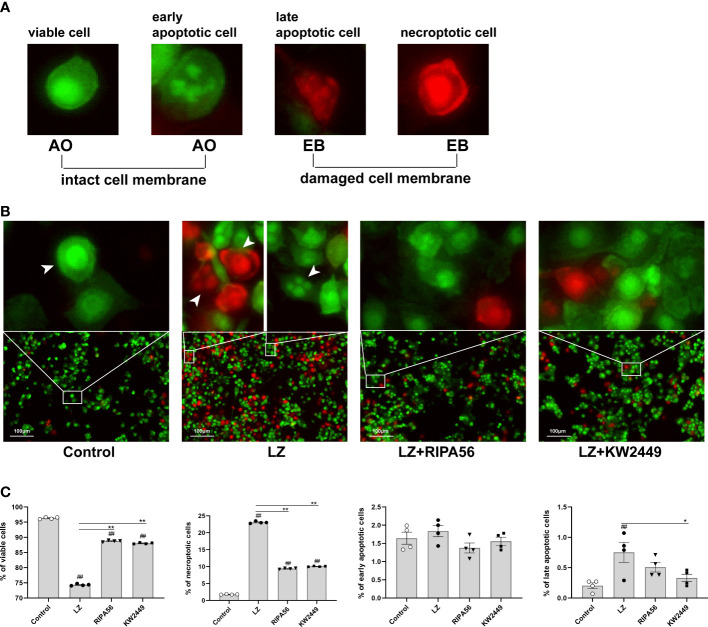
KW2449 reduced LZ-induced necroptosis in RAW 264.7 cells, as detected by AO/EB double staining. **(A)** Typical cell morphology in AO/EB staining. **(B)** Representative images of cell morphological changes in each group after AO/EB staining were observed by a high-content imaging system. Original magnification: 100×, scale bars = 100 μm. **(C)** Data analysis of cell morphology in each group. Means ± SEs, n=4, ^##^ *P <*0.01, vs. control, *P <0.05, **P <0.01, vs. LZ.

Because the use of flow cytometry alone cannot distinguish between late-apoptotic and necroptotic cells, our counting of necroptotic cells inevitably includes with the presence of late-apoptotic cells. To distinguish between different types of cell death, AO/EB staining was performed on the cells, and the morphology and staining of the cells were analyzed by a high-content imaging system (**Figure 5A**). The results showed that necroptosis was the main cause of cell death in the LZ-induced cell model, and the proportion of late-apoptotic cells was low. Therefore, the effect of late-apoptotic cells mixed in necroptotic cell count was negligible when detected by flow cytometry, and the proportion of AV+PI+ cells could appropriately represent the proportion of necroptotic cells in this experiment (**Figures 5A, B**). Moreover, treatment with KW2449 or RIPA56 resulted in increased overall cell viability and decreased cell death, with a significant reduction in the number of necroptotic cells (p<0.01), whereas the number of late-apoptotic cells was too low to be discussed here (**Figures 5B, C**). These results clarify that the inhibition of RIPK1 by KW2449 improves necroptosis rather than apoptosis.

### RIPK1 is an important target that mediates necroptosis and leads to increased inflammation

**Figure 6:**
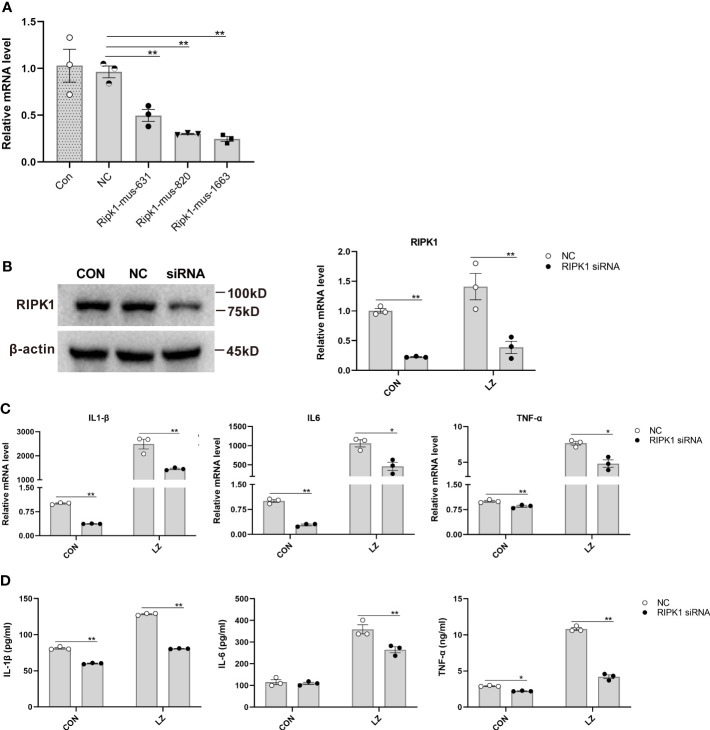
The effect of RIPK1 knockdown on inflammatory cytokines induced by LZ in RAW 264.7 cells. **(A)** The effect of different siRNA sequences on RIPK1 knockdown was measured by qRT−PCR. Means ± SEs, n=3, *P <0.05, **P <0.01, vs. NC (negative control transfected cells). **(B)** RIPK1 knockdown in RAW 264.7 cells with the Ripk1-mus-1663 sequence siRNA was confirmed by Western blot. The low expression of RIPK1 in the LZ model was measured by qRT−PCR. **(C)** The effect of RIPK1 knockdown on IL-6, IL-1β and TNF-α induced by LZ in RAW 264.7 cells was measured by qRT−PCR. **(D)** The effect of RIPK1 knockdown on IL-6, IL-1β and TNF-α induced by LZ in RAW 264.7 cells was detected by ELISA. Means ± SEs, n=3, *P <0.05, **P <0.01, vs. NC.

After confirming the effect of KW2449 on RIPK1 and necroptosis, the role of RIPK1 in the necroptosis pathway was further clarified. Multiple upstream and downstream proteins are involved in the necroptosis pathway, including RIPK1, RIPK3 and MLKL. To further verify whether RIPK1 is essential in this pathway, we used small interfering RNA (siRNA) to knock down RIPK1 and detected the expression of inflammatory cytokines in each group. The results showed that the levels of inflammatory cytokines were increased in the LZ model compared to the control group, and RIPK1 knockdown significantly reduced these increased inflammatory cytokines at both the mRNA and protein levels (p<0.05; p<0.01) (**Figures 6A-D**). These results suggest that RIPK1 is critical for LZ-induced necroptosis and that the use of the RIPK1 inhibitor KW2449 is an effective way to improve the inflammation caused by necroptosis.

## Discussion

In the past few decades, there have been many studies on the pathogenesis of RA, improvements in diagnosis, and the development of new treatment options, which have improved the management of RA and the quality of life and outcomes of RA patients. However, some patients do not respond well to the current treatment options, and the pathogenesis and targeted treatment of RA are still worth exploring (30, 31). Our study found that the plasma levels of RIPK1 and MLKL in RA patients were higher than those in healthy people, which was consistent with the high expression of p-RIPK1 observed in the synovium of RA patients by Patel et al (6). Moreover, their expression levels were found to be positively correlated with the severity of RA (**Figures 3A, B**). Therefore, we suggest that RIPK1 and MLKL have the potential to be novel biomarkers for RA. We further investigated the application of the RIPK1 inhibitor KW2449 in RA treatment and surprisingly found that KW2449 had therapeutic effects in CIA rats. KW2449 is a multikinase inhibitor of (mutant FMS-like tyrosine kinase 3) FLT3, ABL, T315I-mutant ABL (ABL-T315I) tyrosine kinases, Aurora kinase, and fibroblast growth factor receptor (FGFR). It has been used to treat leukemia in previous studies and was later classified as an unclassified RIPK1 inhibitor after its inhibitory effect on RIPK1 was found (18, 32, 33). Given the obvious side effects of MTX in the treatment of RA (34), KW2449 may have the potential to be a new combination or alternative treatment for RA. Our results showed that KW2449 could not only alleviate joint swelling in CIA rats but also reduce joint bone destruction and tissue damage, as well as the plasma levels of the inflammatory cytokines IL-17A, IL-1α, IL-2 and IL-6. These findings suggest that KW2449 can be used as a new potential drug for the treatment of RA, which provides novel insights into treatment for the condition.

Moreover, our study reaffirmed the inhibitory effect of KW2449 on RIPK1 and clarified that RIPK1-mediated necroptosis may play a role in the pathogenesis of RA, which is consistent with the results of previous studies on KW2449 (32). Our results showed that KW2449 not only inhibited the function of RIPK1 to mediate necroptosis, represented by p-RIPK1, but also reduced the overall expression of RIPK1, especially at the protein level. We further distinguished the effect of KW2449 on necroptosis from apoptosis and inflammation, verifying that the effect of KW2449 was specifically on RIPK1-mediated necroptosis. Regarding how necroptosis plays a role in RA, based on our results and the existing research in related fields, we believe that when RIPK1 is activated, MLKL is then activated, resulting in cell membrane perforation and rupture. As a result, a large amount of intracellular content leaks, which induces inflammation in other cells and leads to continuous tissue damage (4–7). Related experiments also found that the second wave of cell death caused by TNF-mediated necroptosis caused stronger and more sustainable expression of inflammatory cytokines than directly mediated inflammation (35). In the LZ cell model, the concomitant reduction in inflammatory cytokines after RIPK1 knockdown also confirmed the importance of RIPK1 in necroptosis-induced inflammation.

Of course, in the process of this research, we also observed some situations that were different from those of previous studies. In previous experiments, RIPK1 deletion caused the death of rats shortly after birth, and the RIPK1 skeleton was assumed to play a protective role (36, 37). However, we believe that this is limited to deletion and does not reflect the effect of excessive RIPK1 *in vivo*. Just as normal immune functions are essential to the body, excessive immunity can still cause damage. Our results showed that excessive RIPK1 expression in CIA rats was accompanied by increased plasma inflammatory cytokines and joint inflammation, both of which decreased after KW2449 treatment, suggesting that excessive RIPK1 expression promoted the inflammatory response in CIA rats. At the same time, the overexpression of RIPK1 synchronously increases the amount of phosphorylated RIPK1, leading to its enhanced function in inducing necroptosis and ultimately increasing the inflammatory response. Therefore, we suggest that, at least in RA, the overexpression of RIPK1 is positively correlated with disease severity. KW2449 not only inhibits RIPK1 phosphorylation but also inhibits the expression of RIPK1, which plays an important role in the treatment of CIA. To clarify these findings, the use of RIPK1 gene knockdown and overexpression in CIA model rats followed by drug treatment will be the direction of our future research. In addition, this experiment lacks a comparison of multidose groups of drugs, so it is not possible to specify the drug dose-effect relationship, which is worthy of further investigation in future experiments. In summary, KW2449 was found to treat CIA rats by inhibiting RIPK1-mediated necroptosis, which was not previously reported. Our study not only provides new ideas for the pathogenesis of RA but also suggests that KW2449, as a small molecule inhibitor targeting RIPK1, has a certain potential in the treatment of RA. It is hoped that with the continuous improvement of research, the mechanism of RA will be clarified, and KW2449 can be applied to the clinic to benefit RA patients.

## Data availability statement

The raw data supporting the conclusions of this article will be made available by the authors, without undue reservation.

## Ethics statement

The studies involving human participants were reviewed and approved by the Ethics Committee of China-Japan Friendship Hospital with the ethical approval number of 2020-133-K86. The patients/participants provided their written informed consent to participate in this study. The animal study was reviewed and approved by the Experimental Animal Ethics Committee of China-Japan Friendship Hospital with the ethical approval number of zryhyy21-21-11-02.

## Author contributions

GZ and CX designed the manuscript. QW wrote the manuscript. QW, QY, XX, XC, XW, MZ, and YX participated in the experiment. TD, XD, GZ, and CX revised the manuscript. All authors contributed to the article and approved the submitted version.
